# The kidney hepcidin/ferroportin axis controls iron reabsorption and determines the magnitude of kidney and systemic iron overload

**DOI:** 10.1016/j.kint.2021.04.034

**Published:** 2021-09

**Authors:** Goran Mohammad, Athena Matakidou, Peter A. Robbins, Samira Lakhal-Littleton

**Affiliations:** 1 Department of Physiology, Anatomy and Genetics, University of Oxford, Oxford, UK; 2 Cancer Research UK Cambridge Institute, University of Cambridge, Cambridge, UK

**Keywords:** ferroportin, hemochromatosis, hepcidin, iron, iron overload, renal tubules

## Abstract

The hepcidin/ferroportin axis controls systemic iron homeostasis by regulating iron acquisition from the duodenum and reticuloendothelial system, respective sites of iron absorption and recycling. Ferroportin is also abundant in the kidney, where it has been implicated in tubular iron reabsorption. However, it remains unknown whether endogenous hepcidin regulates ferroportin-mediated iron reabsorption under physiological conditions, and whether such regulation is important for kidney and/or systemic iron homeostasis. To address these questions, we generated a novel mouse model with an inducible kidney-tubule specific knock-in of fpnC326Y, which encodes a hepcidin-resistant ferroportin termed FPNC326Y. Under conditions of normal iron availability, female mice harboring this allele had consistently decreased kidney iron but only transiently increased systemic iron indices. Under conditions of excess iron availability, male and female mice harboring this allele had milder kidney iron overload, but greater systemic iron overload relative to controls. Additionally, despite comparable systemic iron overload, kidney iron overload occurred in wild type mice fed an iron-loaded diet but not in hemochromatosis mice harboring a ubiquitous knock-in of fpnC326Y. Thus, our study demonstrates that endogenous hepcidin controls ferroportin-mediated tubular iron reabsorption under physiological conditions. It also shows that such control is important for both kidney and systemic iron homeostasis in the context of iron overload.

## Graphical abstract

**Figure:**
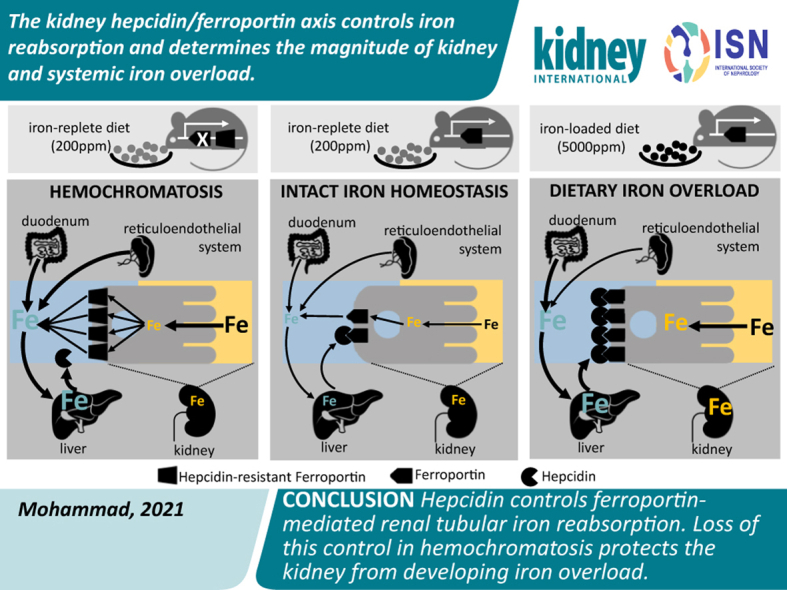

**Translational Statement**

The findings of the present study have implications that must be considered in the management of iron disorders and in the development of new iron therapies. First, strategies targeting the hepcidin/ferroportin (FPN) axis for the treatment of iron overload disorders could affect renal iron levels both directly, by inhibiting renal FPN, and indirectly, by inhibiting FPN in the gut and spleen. Second, increased hepcidin levels in chronic kidney disease could impinge on the progression of renal injury by blocking iron export from renal tubules. Third, the action of hepcidin on renal FPN could modify the outcomes of parenteral iron treatment by promoting iron retention in renal tubules.

Ferroportin (FPN) is the only known mammalian iron export protein. It mediates iron release into the circulation from duodenal enterocytes and splenic reticuloendothelial macrophages, the respective sites of iron absorption and recycling.^1^^,^^2^ FPN-mediated iron release is antagonized by the hormone hepcidin, also known as hepcidin antimicrobial peptide (HAMP). Produced primarily in the liver, hepcidin binds to and induces internalization of FPN, thereby limiting iron release into the circulation and its availability to peripheral tissues.^3^^,^^4^ Thus, the HAMP/FPN axis operates at the sites of absorption and recycling to control systemic iron homeostasis.

The kidney is the site of iron reabsorption. Both non–transferrin-bound and transferrin-bound iron can cross into the glomerular filtrate.^5^ The vast majority of this iron is taken up back into the tubular epithelia. Several transporters have been implicated in this reuptake, including multiligand megalin-cubilin receptor complex, transferrin receptor 1, divalent metal transporter 1, zinc transporter ZIP8, and zinc transporter ZIP14.^5^, ^6^, ^7^, ^8^, ^9^, ^10^

Once in the renal epithelia, iron is reabsorbed into the circulation. FPN is abundant in the kidney and has been implicated in iron reabsorption.^11^, ^12^, ^13^, ^14^ However, it remains unknown if renal FPN is also subject to regulation by endogenous HAMP under normal physiological conditions, and if so, whether such regulation is important for systemic and/or renal iron homeostasis.

To address these questions, we generated a novel mouse model with an inducible renal tubule–specific knock-in of *fpnC326Y*, which encodes a HAMP-resistant FPNC326Y protein. In addition, to confirm the previously reported role of FPN in iron reabsorption, we also generated a mouse model with an inducible renal tubule–specific deletion of the *fpn* gene. Our results demonstrate that endogenous HAMP directly regulates FPN-mediated iron absorption and that this regulation is important for both renal and systemic iron homeostasis, particularly in the setting of excess iron availability.

This study is the first to utilize mice harboring renal-specific loss of HAMP responsiveness to formally determine the importance of the renal HAMP/FPN axis. It provides new insights into the role of iron reabsorption in determining the degree of renal and extrarenal iron overload in the setting of hemochromatosis.

## Methods

### Mice

All animal procedures were compliant with the UK Home Office Animals (Scientific Procedures) Act 1986 and approved by the University of Oxford Medical Sciences Division Ethical Review Committee.

The conditional fpn^fl^ and fpnC326Y^fl^ alleles were generated as described previously.^15^^,^^16^

Mice harboring the Pax8.CreERT2+ transgene were a gift from Dr. Athena Matakidou, Cancer Research UK Cambridge Institute, University of Cambridge. These mice were generated as described previously.^17^ All mice were on a C57BL/6 background.

### Diets

Unless otherwise stated, animals were provided with a standard rodent chow diet containing 200 parts per million (ppm) iron. In iron manipulation experiments, mice were given an iron-loaded diet (5000-ppm iron; Teklad TD.140464) or a matched control diet (200-ppm iron; Teklad TD.08713) from weaning for 3 months.

### Iron quantitation and iron indexes

Serum iron and ferritin levels were determined using the ABX-Pentra system (Horiba Medical). Hemoglobin values were determined by HemoCue Hb 201 Hemoglobin Microcuvettes. Serum erythroferrone levels were measured by enzyme-linked immunosorbent assay (Intrinsic Lifesciences). Determination of total elemental iron in tissues was performed by inductively coupled plasma mass spectrometry, as described previously.^15^^,^^16^^,^^18^ Calibration was achieved using the process of standard additions, where spikes of 0 ng/g, 0.5 ng/g, 1 ng/g, 10 ng/g, 20 ng/g, and 100 ng/g iron were added to replicates of a selected sample. An external iron standard (High Purity Standards ICP-MS-68-A solution) was diluted and measured to confirm the validity of the calibration. Rhodium was also spiked onto each blank, standard, and sample as an internal standard at a concentration of 1 ng/g. For urinary iron quantitation, urine was collected from mice over a period of 24 hours, and subject to inductively coupled plasma mass spectrometry. Creatinine levels in the same samples were measured using a colorimetric creatinine assay kit (Abcam; ab65340), and iron values were normalized to creatinine concentration.

### Immunohistochemistry

Fluorescence immunostaining was performed in formalin-fixed, paraffin-embedded tissue sections, using FPN antibody (Novus Biologicals; NBP1-21502) at 1:100 and Pro-Hepcidin(AA 39-59) antibody at 1:50 (Antibodies Online; ABIN350367). The specificities of the FPN and hepcidin antibodies were confirmed using *Fpn*^fl/fl^, Pax8.CreER^T2+^ animals and Hamp^–/–^ animals as negative controls, respectively (Supplementary Figure S1). Renal segment markers were identified using aquaporin-1 antibody at 1:200 (Biotechne; NB600-749), aquaporin-2 antibody at 1:200 (Biotechne; NBP1-70378), or calbindin antibody at 1:100 (Abcam; ab82812). Secondary antibodies were anti-rabbit IgG Alexa Fluor-488 (Abcam; ab150073), anti-donkey IgG Cy3 (Abcam; ab6949), and anti-mouse IgG Alexa568 (Abcam; ab175473). Slides were imaged using an FV1000 Olympus microscope.

### Western blotting

Tissues were snap frozen in liquid nitrogen, crushed, and then lysed using RIPA Lysis Buffer System (Santa Cruz; sc-24948), according to the manufacturer’s instructions. Tissue lysates were cleared by centrifugation at 15 000*g* for 10 minutes at 4 °C. Protein concentration in the lysates was measured by BCA Protein Assay (Pierce; 23225) and normalized to the same concentration for each batch. Lysates were then diluted in nonreducing Laemmli sodium dodecyl sulfate sample buffer and heated at 95 °C for 5 minutes. Protein (30–50 μg) was loaded onto Mini-PROTEAN TGX Gels (Biorad; 4561096). After electrophoresis, protein was transferred onto polyvinylidene difluoride membrane using the BioRad Translotter system, and membranes were blocked for an hour in blocking buffer containing 5% bovine serum albumin. Membranes were then stained overnight at 4 °C with rabbit polyclonal anti-mouse FPN antibody (NBP1-21502; Novus Biologicals) at 1:1000 or horseradish peroxidase–conjugated anti–β-actin antibody (Proteintech; HRP-60008) at 1:5000. Blots were developed using the ECL prime detection kit (RPN2232; VWR International). Signal intensities were quantified by ImageJ, and the ratio between the FPN and the β-actin signals was calculated to produce normalized intensities.

### Diaminobenzidine (DAB)-enhanced Perls stain

Formalin-fixed, paraffin-embedded tissue sections were deparaffinized using xylene, and then rehydrated in ethanol. Slides were then stained for 1 hour with 1% potassium ferricyanide in 0.1 mol/L HCl buffer. Endogenous peroxidase activity was quenched, and then slides were stained with DAB chromogen substrate and counterstained with hematoxylin. They were visualized using a standard bright-field microscope.

### Quantitative Polymerase Chain Reaction

Gene expression was measured using Applied Biosystems TaqMan gene expression assay probes for *Hamp* and housekeeping gene *β-actin* (Life Technologies). The threshold cycle (CT) value for the gene of interest was first normalized by deducting CT value for *β-actin* to obtain a ΔCT value. ΔCT values of test samples were further normalized to the average of the ΔCT values for control samples to obtain ΔΔCT values. Relative gene expression levels were then calculated as 2^–ΔΔCT^.

### Statistics

Values are shown as mean ± SEM. Paired comparisons were performed using the Student *t* test. Multiple comparisons were drawn using analysis of variance. *Post hoc* tests used Bonferroni correction.

## Results

### The hepcidin/FPN axis in renal tubules controls iron reabsorption and is important for renal iron homeostasis in female mice

**Figure 1:**
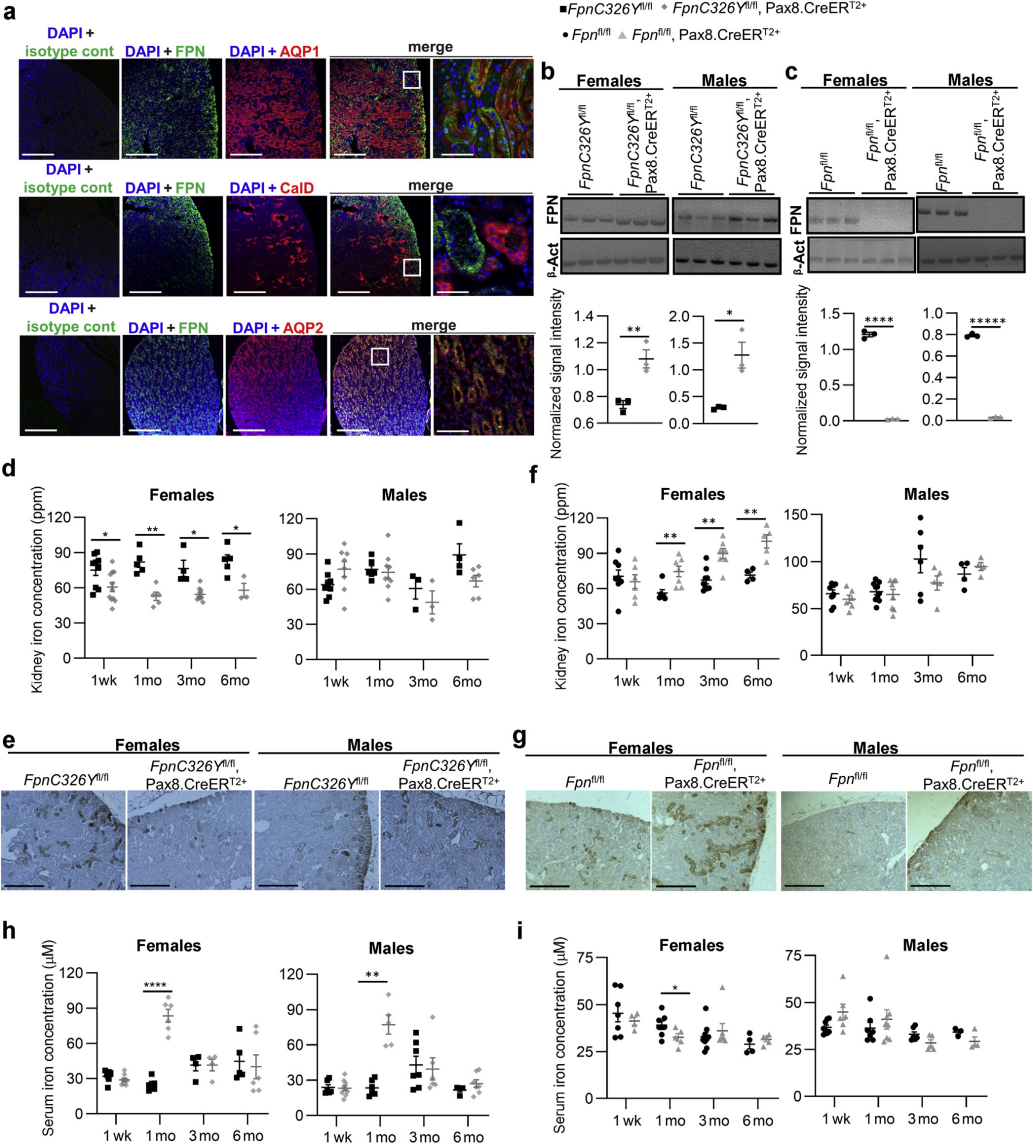
**The hepcidin/ferroportin (FPN) axis in renal tubules controls iron reabsorption and is important for renal iron homeostasis in female mice.** (**a**) Representative images of immunofluorescent staining for FPN, aquaporin 1 (AQP1), aquaporin 2 (AQP2), and Calbindin (CalD) in kidneys of wild-type mice. Bars = 200 μm, original magnification ×10. End panel, bar = 25 μm, original magnification ×60. (**b**) Western blot for FPN in kidneys of female and male *FpnC326Y*^fl/fl^,Pax8.CreER^T2+^ mice and *FpnC326Y*^fl/fl^ controls at 1 week after tamoxifen treatment. Quantitation of signal intensity shown in bottom panel. (**c**) Western blot for FPN in kidneys of female and male *Fpn*^fl/fl^,Pax8.CreER^T2+^ mice and *Fpn*^fl/fl^ controls at 1 week after tamoxifen treatment. Quantitation of signal intensity shown in bottom panel. (**d**) Renal iron levels in female and male *FpnC326Y*^fl/fl^,Pax8.CreER^T2+^ mice and *FpnC326Y*^fl/fl^ controls at 1 week, 1 month, 3 months, and 6 months after tamoxifen treatment. (**e**) Representative images of diaminobenzidine (DAB)-enhanced Perls iron stain in the renal cortical region of corresponding animals at 3 months after tamoxifen induction. Bars = 200 μm, original magnification ×10. (**f**) Renal iron levels in female and male *Fpn*^fl/fl^,Pax8.CreER^T2+^ mice and *Fpn*^fl/fl^ controls at 1 week, 1 month, 3 months, and 6 months after tamoxifen induction. (**g**) Representative images of DAB-enhanced Perls iron stain in the renal cortical region of corresponding animals at 3 months after tamoxifen induction. Bars = 200 μm, original magnification ×10. (**h**) Serum iron levels in female and male *FpnC326Y*^fl/fl^,Pax8.CreER^T2+^ mice and *FpnC326Y*^fl/fl^ controls at 1 week, 1 month, 3 months, and 6 months after tamoxifen treatment. (**i**) Serum iron levels in female and male *Fpn*^fl/fl^,Pax8.CreER^T2+^ mice and *Fpn*^fl/fl^ controls at 1 week, 1 month, 3 months, and 6 months after tamoxifen induction. Values are shown as mean ± SEM. ∗*P* < 0.05, ∗∗*P* < 0.01, and ∗∗∗∗*P* < 0.0001. β-Act, β-Actin; cont, control; DAPI, 4′,6-diamidino-2-phenylindole; ppm, parts per million. To optimize viewing of this image, please see the online version of this article at www.kidney-international.org.

We first sought to establish the exact site of FPN expression in the kidney. To that end, we costained mouse kidneys for FPN and segment-specific markers aquaporin 1 (a marker of proximal convoluted tubules and thin descending limb of Henley), aquaporin 2 (a marker of collecting ducts and connecting tubules), and calbindin (a marker of distal convoluted tubules and cortical connecting and collecting ducts). We found that FPN is strongly expressed in the cortex, colocalizing with aquaporin 1 to the proximal convoluted tubules. There was some FPN in the inner medulla, colocalizing with aquaporin 2 to the medullary collecting ducts. There was no colocalization of FPN with calbindin (Figure 1a; larger panel shown in Supplementary Figure S2). These results confirm that FPN is most abundant in the cortical region within proximal convoluted tubules.

To determine whether renal FPN is regulated by HAMP, we used mice harboring a Pax8.CreER^T2+^ knock-in transgene, which drives tamoxifen-inducible expression of the Cre recombinase under control of the paired box gene 8 *pax8* promoter in proximal and distal tubules and in collecting ducts.^17^ We crossed Pax8.CreER^T2+^ mice with those harboring a conditional knock-in floxed allele *FpnC326Y*, which encodes a hepcidin-resistant FPN. In addition, and to confirm the previously reported role of renal FPN in iron reabsorption, we crossed Pax8.CreER^T2+^ mice with mice harboring the *Fpn*^fl/fl^ allele. One week following tamoxifen treatment to induce the Pax8.CreER^T2^ transgene, renal FPN levels were increased in female and male *FpnC326Y*^fl/fl^,Pax8.CreER^T2+^ mice relative to *FpnC326Y*^fl/fl^ controls (Figure 1b), demonstrating that renal FPN is subject to regulation by endogenous HAMP under normal physiological conditions, and in addition confirming the efficiency of the Pax8.CreER^T2+^ transgene. Conversely, renal FPN levels were reduced in female and male *Fpn*^fl/fl^,Pax8.CreER^T2+^ mice relative to *Fpn*^fl/fl^ controls, further confirming the efficiency of the Pax8.CreER^T2+^ transgene (Figure 1c). The specificity of this Pax8.CreER^T2+^ transgene was also confirmed by the presence of the deletion allele (ΔFpn) in the kidney but not in the liver or spleen of *Fpn*^fl/fl^,Pax8.CreER^T2+^ mice (Supplementary Figure S2A).

Next, we set out to determine whether the renal HAMP/FPN axis controls iron reabsorption. To that end, we measured renal and serum iron levels at 1 week, 1 month, 3 months, and 6 months after tamoxifen treatment. We found that renal iron content was lower in *FpnC326Y*^fl/fl^,Pax8.CreER^T2+^ females than in *FpnC326Y*^fl/fl^ control females at all time points (Figure 1d). Renal iron content in males was not different according to genotype (Figure 1d). DAB-enhanced Perls iron stain also confirmed the reduction in iron levels within proximal tubules of *FpnC326Y*^fl/fl^,Pax8.CreER^T2+^ females (Figure 1e; larger panel shown in Supplementary Figure S2). Conversely, we found that renal iron content was higher in *Fpn*^fl/fl^,Pax8.CreER^T2+^ females than in *Fpn*^fl/fl^ control females from 1 month onwards (Figure 1f). Renal iron content in males was not different according to genotype (Figure 1f). DAB-enhanced Perls iron stain also confirmed iron accumulation within proximal tubules of *Fpn*^fl/fl^,Pax8.CreER^T2+^ females (Figure 1g; larger panel shown in Supplementary Figure S2).

Serum iron levels were increased transiently in both male and female *FpnC326Y*^fl/fl^,Pax8.CreER^T2+^ mice relative to *FpnC326Y*^fl/fl^ controls at the 1-month time point (Figure 1h). Conversely, serum iron levels were decreased in *Fpn*^fl/fl^,Pax8.CreER^T2+^ females relative to *Fpn*^fl/fl^ control females at the 1-month time point, whereas they remained comparable in males of different genotypes at all time points (Figure 1i). Restoration of serum iron levels to those seen in control animals at 3 months of age could not be attributed to restoration of normal renal FPN levels. Indeed, the Pax8.CreER^T2+^-induced changes in renal FPN levels were still maintained at 3 months after tamoxifen treatment (Supplementary Figure S2B and C). Decreased renal iron content and increased serum iron levels in *FpnC326Y*^fl/fl^,Pax8.CreER^T2+^ females demonstrate that FPN-dependent iron reabsorption in proximal tubules is subject to regulation by HAMP under normal physiological conditions. Increased renal iron content and decreased serum iron levels in *Fpn*^fl/fl^,Pax8.CreER^T2+^ females confirm previous findings that renal FPN contributes to iron reabsorption. In addition, under normal physiological conditions, the control of iron reabsorption by the renal HAMP/FPN axis appears to be more important in females than in males, at least in the C57BL/6 strain.

### The renal HAMP/FPN axis contributes to, but is not essential for, normal systemic iron homeostasis under conditions of normal iron availability

**Figure 2:**
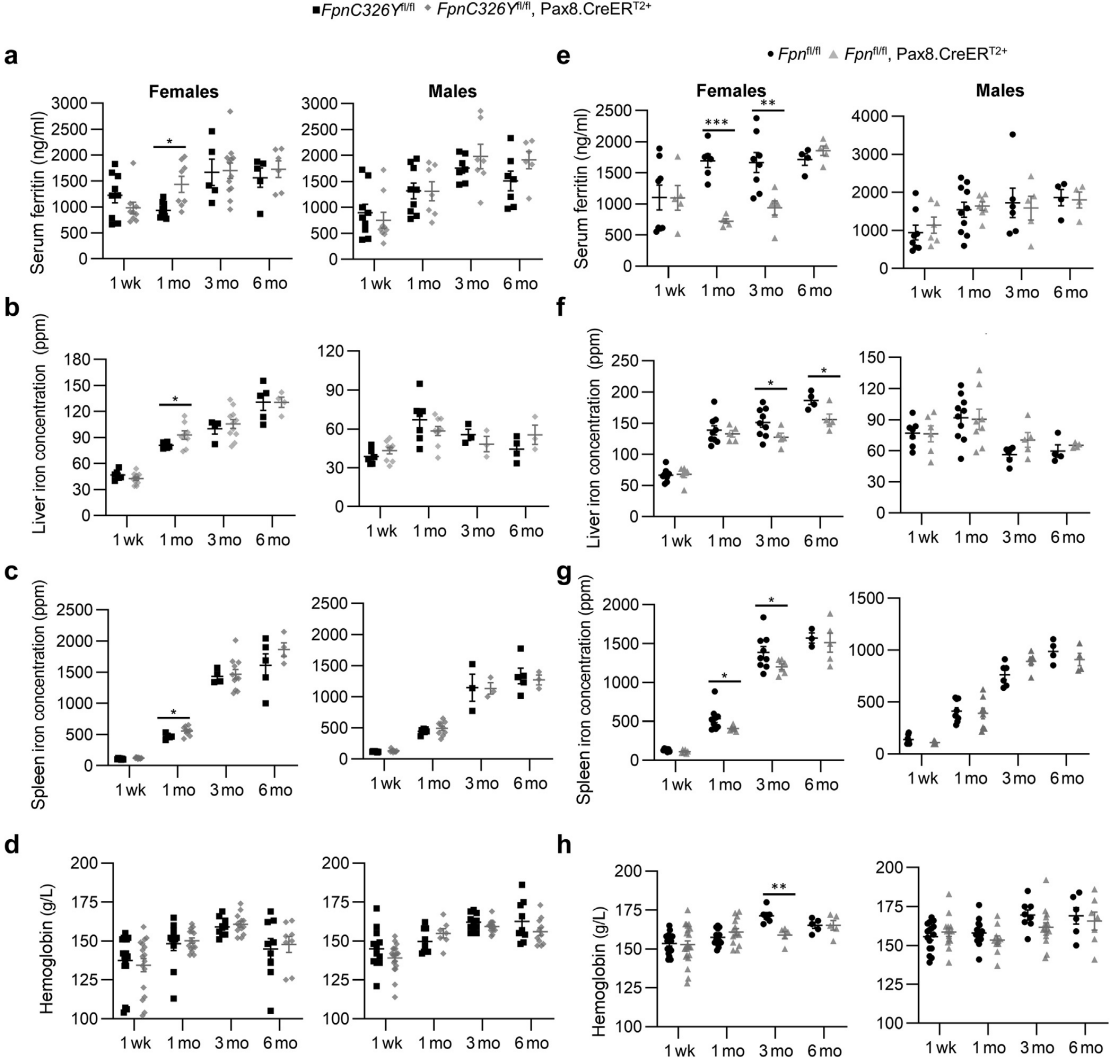
**The renal hepcidin antimicrobial peptide/ferroportin (FPN) axis contributes to, but is not essential for, normal systemic iron homeostasis under conditions of normal iron availability.** (**a**–**d**) Systemic iron indexes in female and male *FpnC326Y*^fl/fl^,Pax8.CreER^T2+^ mice and *FpnC326Y*^fl/fl^ controls at 1 week, 1 month, 3 months, and 6 months after tamoxifen treatment, including (**a**) serum ferritin, (**b**) liver iron content, (**c**) spleen iron content, and (**d**) hemoglobin. (**e**–**h**) Systemic iron indexes in female and male *Fpn*^fl/fl^,Pax8.CreER^T2+^ mice and *Fpn*^fl/fl^ controls at 1 week, 1 month, 3 months, and 6 months after tamoxifen induction, including (**e**) serum ferritin, (**f**) liver iron content, (**g**) spleen iron content, and (**h**) hemoglobin. Values are shown as mean ± SEM. ∗*P* < 0.05, ∗∗*P* < 0.01, and ∗∗∗*P* < 0.001. ppm, parts per million.

Next, we set out to determine the contribution of the renal HAMP/FPN axis to systemic iron homeostasis. We found that *FpnC326Y*^fl/fl^,Pax8.CreER^T2+^ females had a transient increase in serum ferritin (Figure 2a), liver iron content (Figure 2b), and spleen iron content (Figure 2c) at the 1-month time point when compared with *FpnC326Y*^fl/fl^ control females. Their hemoglobin levels remained comparable to those of controls at all time points (Figure 2d). None of these parameters was different between *FpnC326Y*^fl/fl^,Pax8.CreER^T2+^ males and their *FpnC326Y*^fl/fl^ controls (Figure 2a–d). Erythroferrone levels were not different according to genotype (Supplementary Figure 3A). Conversely, we found that *Fpn*^fl/fl^,Pax8.CreER^T2+^ females have lower serum ferritin levels at the 1- and 3-month time points (Figure 2e), lower liver iron content at the 3- and 6-month time points (Figure 2f), and lower spleen iron content at the 1- and 3-month time points (Figure 2g), when compared with *Fpn*^fl/fl^ control females. They also had a transient and mild reduction in hemoglobin levels at the 3-month time point (Figure 2h). None of these parameters was different between *Fpn*^fl/fl^,Pax8.CreER^T2+^ males and their *Fpn*^fl/fl^ controls (Figure 2e–h). Erythroferrone levels were also not altered according to genotype (Supplementary Figure 3B). Together, these data demonstrate that, under normal physiological conditions, the renal HAMP/FPN axis contributes to systemic iron levels but in itself is not essential for the maintenance of normal systemic iron homeostasis.

### The renal HAMP/FPN axis determines the magnitude of renal and systemic iron overload under conditions of excess iron availability

**Figure 3:**
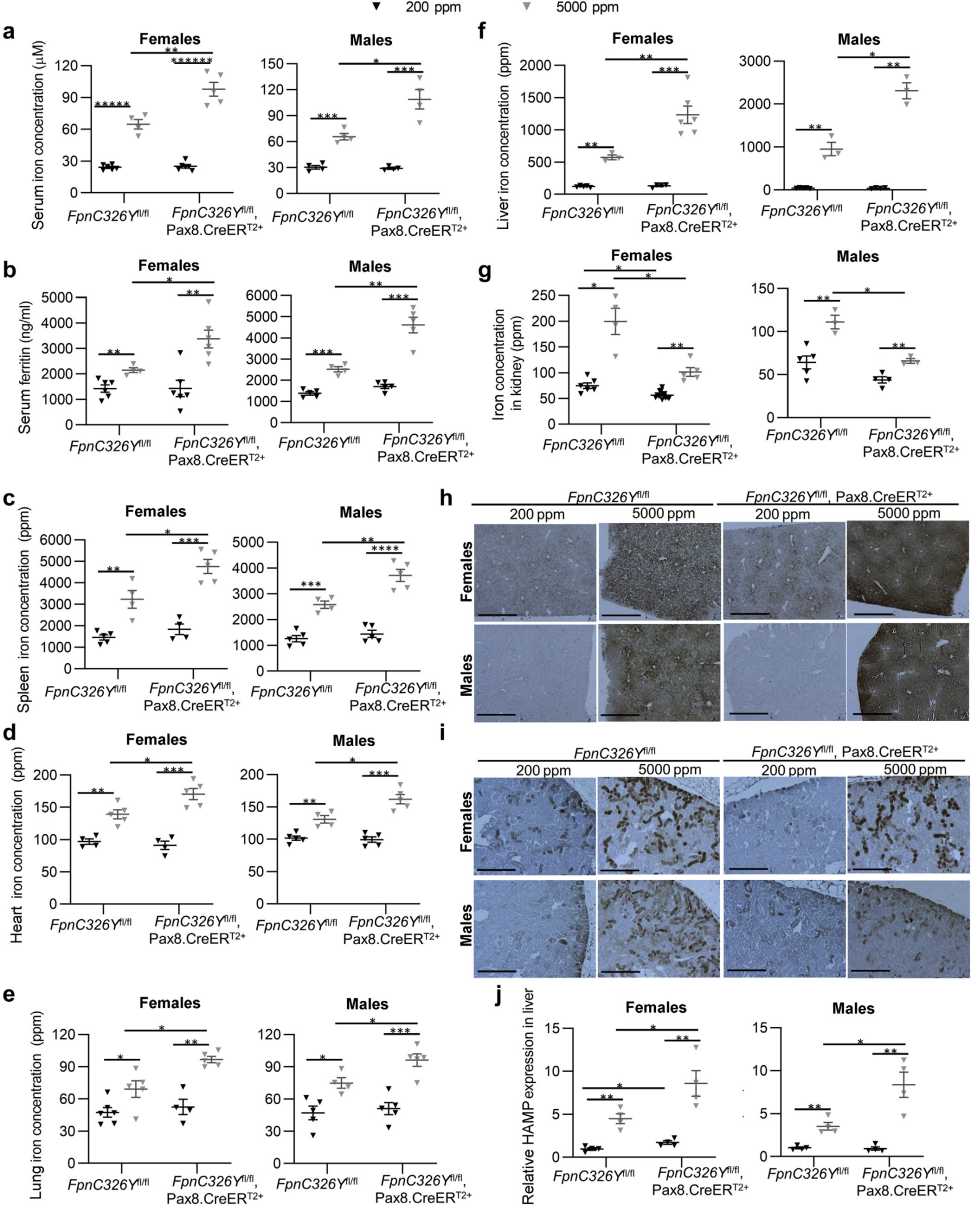
**The renal hepcidin antimicrobial peptide (HAMP)/ferroportin (FPN) axis determines the magnitude of renal and systemic iron overload under conditions of excess iron availability.** (**a**–**f**) Extrarenal iron indexes in female and male *FpnC326Y*^fl/fl^,Pax8.CreER^T2+^ mice and *FpnC326Y*^fl/fl^ controls provided either a control chow diet (200 parts per million [ppm]) or an iron-loaded diet (5000 ppm) for 3 months. Indexes include (**a**) serum iron, (**b**) serum ferritin, (**c**) spleen iron content, (**d**) heart iron content, (**e**) lung iron content, and (**f**) liver iron content. (**g**) Renal iron content in female and male *FpnC326Y*^fl/fl^,Pax8.CreER^T2+^ mice and *FpnC326Y*^fl/fl^ controls provided either a control chow diet (200 ppm) or an iron-loaded diet (5000 ppm) for 3 months. (**h,i**) Representative images of diaminobenzidine (DAB)-enhanced Perls iron stain in livers and the renal critical region of corresponding animals. Bars = 200 μm, original magnification ×10. (**j**) Relative expression of the *hamp* gene in the livers of corresponding animals. Values are shown as mean ± SEM. ∗*P* < 0.05, ∗∗*P* < 0.01, ∗∗∗*P* < 0.001, ∗∗∗∗*P* < 0.0001, ∗∗∗∗∗*P* < 0.00001, and ∗∗∗∗∗∗*P* < 0.000001. To optimize viewing of this image, please see the online version of this article at www.kidney-international.org.

Next, we set out to determine the role of the renal HAMP/FPN axis in the setting of iron overload. To that effect, *FpnC326Y*^fl/fl^,Pax8.CreER^T2+^ animals and *FpnC326Y*^fl/fl^ controls were induced with tamoxifen and then provided either a control chow diet, containing 200 ppm iron, or an iron-loaded diet, containing 5000 ppm iron, for 3 months. We found that provision of iron-loaded diet increased serum iron, serum ferritin, and tissue iron content in males and females of both genotypes. However, *FpnC326Y*^fl/fl^,Pax8.CreER^T2+^ animals had greater increase in serum iron (Figure 3a), serum ferritin (Figure 3b), and iron content in the spleen (Figure 3c), heart (Figure 3d), lung (Figure 3e), and liver (Figure 3f) than *FpnC326Y*^fl/fl^ controls. In contrast, they had a lower increase in renal iron content (Figure 3g). Differences between genotypes in the degree of iron loading within proximal tubules and in the liver were also apparent in DAB-enhanced Perls iron stain (Figure 3h and i; larger panels shown in Supplementary Figure S4). In line with greater systemic iron overload, *FpnC326Y*^fl/fl^,Pax8.CreER^T2+^ animals also had greater increase in liver *hamp* gene expression compared with *FpnC326Y*^fl/fl^ controls (Figure 3j). Hemoglobin levels were not affected by either diet or genotype, and consistent with this, serum erythroferrone levels also remained unchanged (Supplementary Figure 4A and B). These findings demonstrate that, under conditions of excess iron availability, control of iron reabsorption by the renal HAMP/FPN axis decreases systemic iron overload while increasing renal iron overload.

### The renal HAMP/FPN axis determines the pattern of tissue iron overload in hemochromatosis

**Figure 4:**
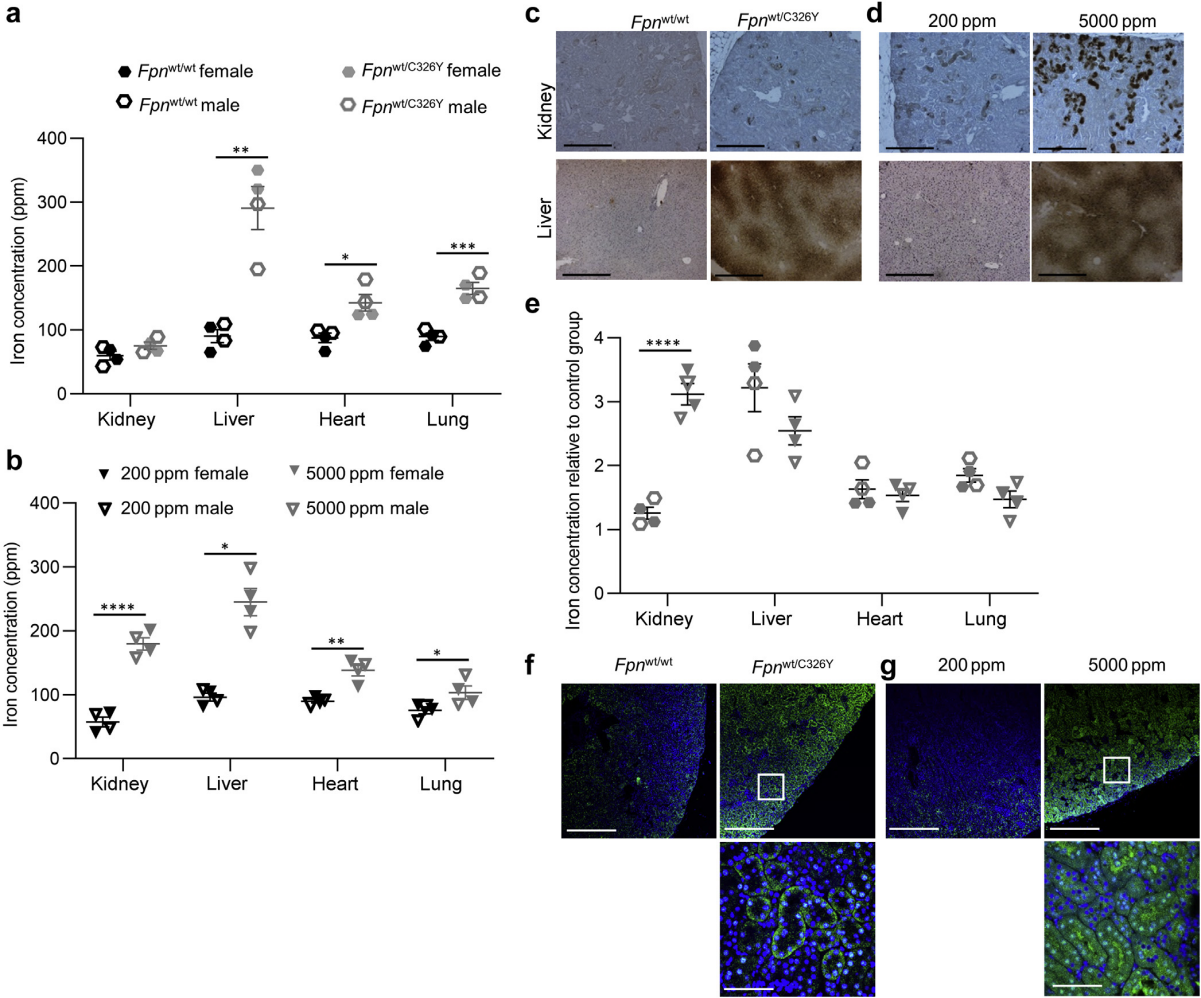
**The renal hepcidin antimicrobial peptide/ferroportin (FPN) determines the pattern of tissue iron overload in hemochromatosis.** (**a**) Iron levels in the kidney, liver, heart, and lung of *Fpn*^wt/C326Y^ animals and *Fpn*^wt/wt^ controls at 3 months of age. (**b**) Iron levels in the kidney, liver, heart, and lung in wild-type animals provided either a control chow diet (200 parts per million [ppm]) or an iron-loaded diet (5000 ppm) from weaning for 3 months. (**c**) Representative images of diaminobenzidine (DAB)-enhanced Perls iron stain in the renal cortex and livers of *Fpn*^wt/C326Y^ animals and *Fpn*^wt/wt^ controls at 3 months of age. Bar = 200 μm, original magnification ×10. (**d**) Representative images of DAB-enhanced Perls iron stain in the renal cortex and livers of wild-type animals provided either a control chow diet (200 ppm) or an iron-loaded diet (5000 ppm) from weaning for 3 months. Bar = 200 μm, original magnification ×10. (**e**) Comparison of the degree of tissue iron loading between *Fpn*^wt/C326Y^ animals and wild-type animals fed an iron-loaded diet. Values shown for each animal are normalized to the mean of the respective control group. (**f**) Representative images of FPN immunofluorescent staining in the renal cortex of *Fpn*^wt/C326Y^ animals and *Fpn*^wt/wt^ controls at 3 months of age. Top panel, bars = 200 μm, original magnification ×10. Bottom panel, bar = 25 μm, original magnification ×60. (**g**) Representative images of FPN immunofluorescent staining in the renal cortex of wild-type animals provided either a control chow diet (200 ppm) or an iron-loaded diet (5000 ppm) from weaning for 3 months. Top panel, bars = 200 μm, original magnification ×10. Bottom panel, bar = 25 μm, original magnification ×60. Values are shown as mean ± SEM. ∗*P* < 0.05, ∗∗*P* < 0.01, ∗∗∗*P* < 0.001, and ∗∗∗∗*P* < 0.0001. To optimize viewing of this image, please see the online version of this article at www.kidney-international.org.

Next, we set out to explore the role of the renal HAMP/FPN axis in the context of hereditary hemochromatosis, a genetic condition of iron overload caused by defects in hepcidin production or hepcidin responsiveness.^19^ To that effect, we used mice generated in-house harboring a heterozygous ubiquitous knock-in of the fpnC326Y allele (*Fpn*^wt/C326Y^). We had previously demonstrated that these mice develop the iron-overload phenotype characteristic of hereditary hemochromatosis.^15^^,^^18^ We compared the pattern of tissue iron overload in these mice with that seen in wild-type mice fed an iron-loaded diet from weaning for 3 months. Both *Fpn*^wt/C326Y^mice and wild-type mice fed an iron-loaded diet had increased iron content in the liver, heart, and lung compared with their respective controls (Figure 4a and b). Renal iron content in *Fpn*^wt/C326Y^mice was normal at 3 months of age (Figure 4a) and only increased by 32% relative to controls at 6 months of age (Supplementary Figure 5A). In contrast, wild-type animals provided an iron-loaded diet had a 260% increase in renal iron content compared with those on a normal diet (Figure 4b). Relative differences in renal and liver iron overload between the 2 models were also apparent in DAB-enhanced Perls iron stain (Figure 4c and d; larger panels shown in Supplementary Figure S5). The magnitude of extrarenal iron overload was comparable between the 2 models, whereas the magnitude of renal overload was considerably higher in wild-type animals provided an iron-loaded diet than in *Fpn*^wt/C326Y^ mice (Figure 4e). FPN was increased in the cortex in both *Fpn*^wt/C326Y^ mice and wild-type mice provided an iron-loaded diet (Figure 4f and g; larger panels shown in Supplementary Figure S5). Closer examination of the site of FPN expression in proximal tubules revealed that it appeared to localize intracellularly as well as to the basolateral membrane in *Fpn*^wt/C326Y^ mice. In contrast, FPN appeared to localize primarily to the cytoplasm, apical membrane, and surprisingly the nucleus in mice provided an iron-loaded diet (Figure 4f and g, lower panels). The pattern of FPN localization arising from the provision of iron-loaded diet was further confirmed using *Fpn*^fl/fl^,Pax8.CreER^T2+^ *Fpn*^fl/fl^,Pax8.CreER^T2+^ animals as negative controls (Supplementary Figure S5B). These observations, together with the previous finding that *FpnC326Y*^fl/fl^,Pax8.CreER^T2+^ animals have lower renal iron loading than controls following provision of an iron-loaded diet, demonstrate that loss of HAMP action on renal FPN protects the kidney from iron loading in the setting of hereditary hemochromatosis.

## Discussion

The most important finding of the present study is that endogenous HAMP controls FPN-mediated iron reabsorption. Previous studies had reported the regulation of FPN by exogenous HAMP in cultured renal cells, and an inverse relationship between HAMP and FPN levels in the kidney following unilateral ureter occlusion.^12^^,^^13^ However, this is the first demonstration that such regulation operates *in vivo* under normal physiological conditions, and in a manner that impinges on renal and systemic iron homeostasis. A particular strength of this study is the use of novel mice, generated in-house, to harbor a renal-specific loss of HAMP responsiveness. This approach allows the study of the renal HAMP/FPN axis without the confounding effects of altered systemic iron homeostasis, otherwise seen in ubiquitous animal models.

**Figure 5:**
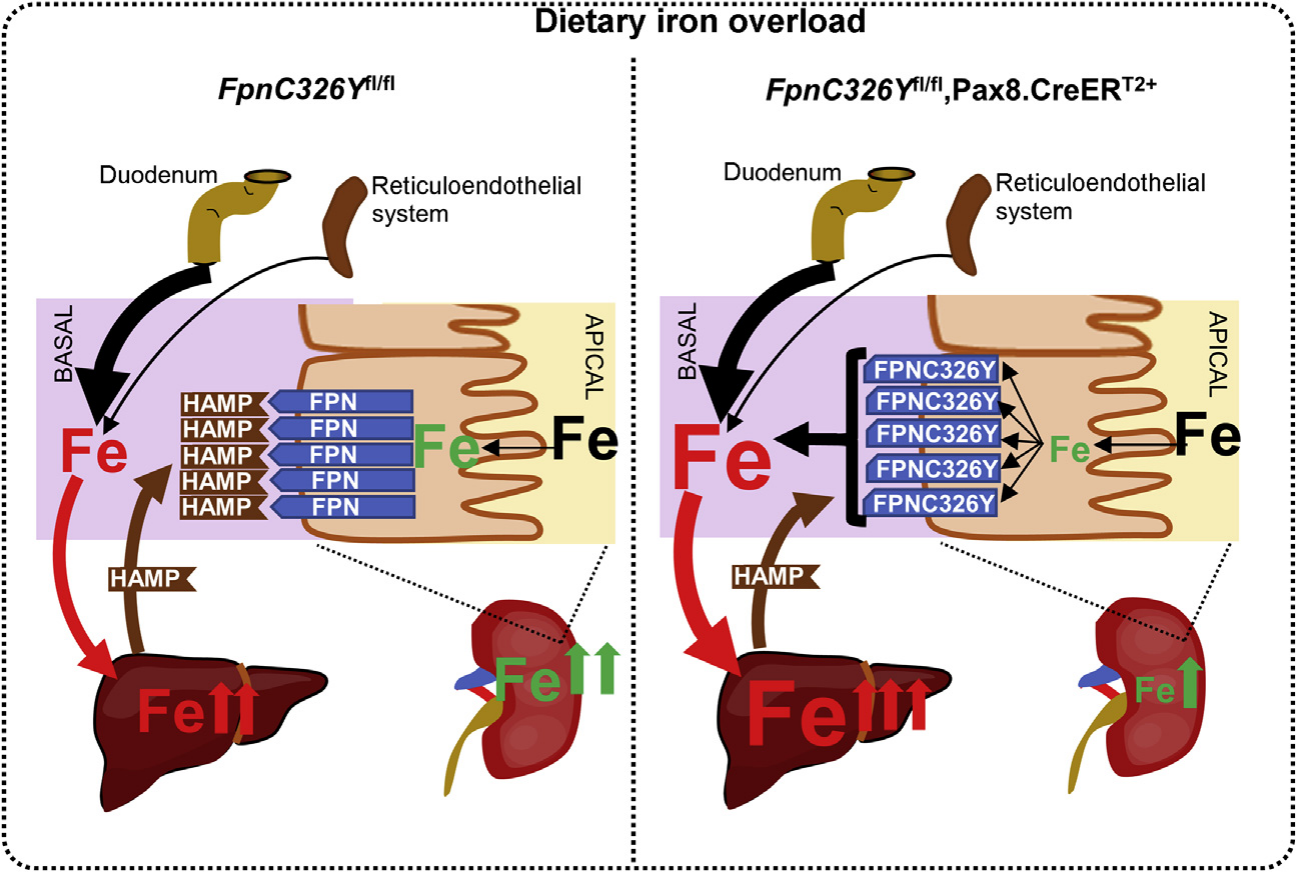
**The role of the renal hepcidin antimicrobial peptide (HAMP)/ferroportin (FPN) axis in the setting of iron overload.** Provision of iron-loaded diet increases serum iron availability and iron levels in the glomerular filtrate and increases hepcidin (HAMP) production and release by the liver. Increased serum HAMP inhibits iron reabsorption by blocking FPN in renal tubules. Inhibition of iron reabsorption causes renal iron retention while decreasing systemic iron availability and consequently reducing liver iron overload. In *FpnC3326Y*^fl/fl^,Pax8.CreER^T2+^ mice, the loss of HAMP responsiveness in the renal tubules causes unregulated iron reabsorption. This, in turn, prevents renal iron retention while increasing systemic iron availability and subsequently increasing liver iron overload.

The second important finding of the present study is that the renal HAMP/FPN determines the magnitude of both renal and systemic iron overload (Figure 5). Indeed, loss of HAMP responsiveness in renal tubules increased the magnitude of liver, spleen, heart, and lung iron overload, while reducing the magnitude of renal iron overload following provision of an iron-loaded diet. Consistent with this, greater renal iron overload was seen following provision of an iron-loaded diet to wild-type animals (in which the renal HAMP/FPN axis is intact) than in hemochromatosis mice (in which the renal HAMP/FPN axis is also disrupted) (graphical abstract). This finding may explain the clinical observation that the kidney is not commonly affected in patients with hereditary hemochromatosis.^19^

Although both hemochromatosis and dietary iron overload increased FPN in renal tubules, they appeared to result in different patterns of localization to the apical, intracellular, or basolateral compartments. Similar observations were made in the rat duodenum, where iron gavage was found to alter the relative distribution of FPN between these compartments in duodenal enterocytes.^20^ One interpretation of these results is that in the setting of dietary iron overload, increased FPN translation (mediated by iron regulatory proteins) would act to maintain high overall FPN production in the renal tubular cell, whereas increased serum HAMP would reduce the proportion of FPN that can localize to the basolateral membrane. It follows from this interpretation that the divergence between previous reports as to the localization of FPN in proximal tubules could reflect differences in renal tubular iron content and in serum hepcidin levels between animal models used in different studies.^5^^,^^11^, ^12^, ^13^, ^14^^,^^21^ Alternatively, this divergence could reflect a degree of nonspecific reactivity reported previously in relation to commercial FPN antibodies, including the one used in the present study.^14^ The functional significance of the apical localization of FPN in renal tubules remains unclear, and we could not detect any changes in urinary iron excretion in iron-loaded mice (Supplementary Figure 5C). Another interesting observation is that some FPN appeared localized to the nucleus in renal tubules of iron-loaded mice. Similar observations were made by others in iron-loaded macrophages and in the rat liver. The latter study found nuclear FPN to be involved in nuclear iron retention as part of the acute phase response.^22^^,^^23^ Detailed studies of the subcellular localization of FPN in different iron states and in different pathologies are needed to further advance our understanding of the role of FPN in the kidney.

The regulation and function of renal HAMP are also not completely understood. We found that expression of the *hamp* gene in the kidney was increased in wild-type animals following provision of iron-loaded diet (Supplementary Figure 5E), but remained unaltered in hemochromatosis mice (Supplementary Figure 5D), and in those harboring renal tubule–specific loss of FPN or renal tubule–specific loss of HAMP responsiveness (Supplementary Figure S5F and G). These results do not support the notion that renal *hamp* gene expression is regulated locally by renal iron levels. In terms of the function of renal HAMP, a previous study in a model of unilateral ureter occlusion suggested that it is involved in controlling renal FPN. We found that renal HAMP (detected using an antibody against pro-HAMP peptide) colocalized with calbindin (a marker of distal convoluted tubules and cortical collecting ducts and connecting ducts) (Supplementary Figure S5H). On the other hand, FPN expression does not colocalize with calbindin, even in mice harboring renal-specific loss of HAMP responsiveness (Figure 1a and Supplementary Figure S5I). These results are not consistent with an autocrine role for renal HAMP in the regulation of renal FPN. Nonetheless, renal HAMP may be involved in paracrine regulation of renal FPN. In the future, it would be interesting to study the paracrine functions of renal HAMP, and to quantify the relative contributions of renal and hepatic HAMPs to the control of iron reabsorption.

Another interesting observation from the present study is that, under conditions of normal iron availability, the renal HAMP/FPN axis is not essential for maintenance of normal systemic iron homeostasis and is instead more important for regulating renal iron levels, at least in female mice of the C57BL/6 strain. Indeed, although the observed changes in renal iron levels resulting from loss of FPN or of HAMP responsiveness in the renal tubules were persistent, changes in systemic iron indexes were transient in nature. The transient nature of the systemic effects suggests the involvement compensatory mechanism(s). One possible compensatory mechanism is modulation of dietary iron absorption by hepatic HAMP. Indeed, we found that in mice harboring renal-specific loss of HAMP responsiveness, hepatic *hamp* gene expression was increased following the increase in serum iron levels at the 1-month time point and remained increased at later time points. Conversely, hepatic *hamp* gene expression was suppressed in mice harboring renal-specific loss of FPN following the decrease in serum iron levels at the 1-month time point and remained suppressed at later time points (Supplementary Figure S6A and B). In addition, increased hepatic *hamp* gene expression in the first setting was accompanied by decreased gut FPN levels, whereas decreased hepatic *hamp* gene expression in the second setting was accompanied by higher gut FPN (Supplementary Figure S6C and D). These results indicate that the action of hepatic HAMP on dietary iron absorption is an active compensatory mechanism involved in maintaining normal systemic iron homeostasis in the face of perturbed renal iron reabsorption.

Of note, under conditions of normal iron availability, the renal HAMP/FPN axis appears to be more important in female mice that in male mice, at least in the C57BL/6 strain. This observation could not be attributed to differences between males and females in the activity of the Pax8.CreER^T2+^ transgene. Sexual dimorphism in the levels and patterns of transporters along the nephron has been reported previously in this mouse strain.^24^ Consistent with this, we found higher basal expression of FPN in the kidneys of females than in the kidneys of male littermates (Supplementary Figure S2D). A previous study using a different Cre recombinase transgene, driven by a constitutively active Nestin promoter to delete *fpn* in the entire nephron, also reported an increase in renal iron levels, and decrease in serum iron and liver iron stores. However, that study was conducted in a different mouse strain (129/SvEvTac) and did not analyze the phenotype according to sex or time course.^14^

Together, the findings of the present study provide new understanding of the role of the renal HAMP/FPN axis in renal and systemic iron homeostasis. This new understanding has potentially important implications for the management of hemochromatosis and other iron disorders.

## Disclosure

All the authors declared no competing interests.
