# Supplementary material for: The kidney hepcidin/ferroportin axis controls iron reabsorption and determines the magnitude of kidney and systemic iron overload
Source: Kidney Int. 2021 Sep;100(3):559–69. doi: 10.1016/j.kint.2021.04.034 (PMC8456337; doi:10.1016/j.kint.2021.04.034)
Supplement: Supplementary File (PDF) [file mmc1.pdf]

Supplemental Figure 1

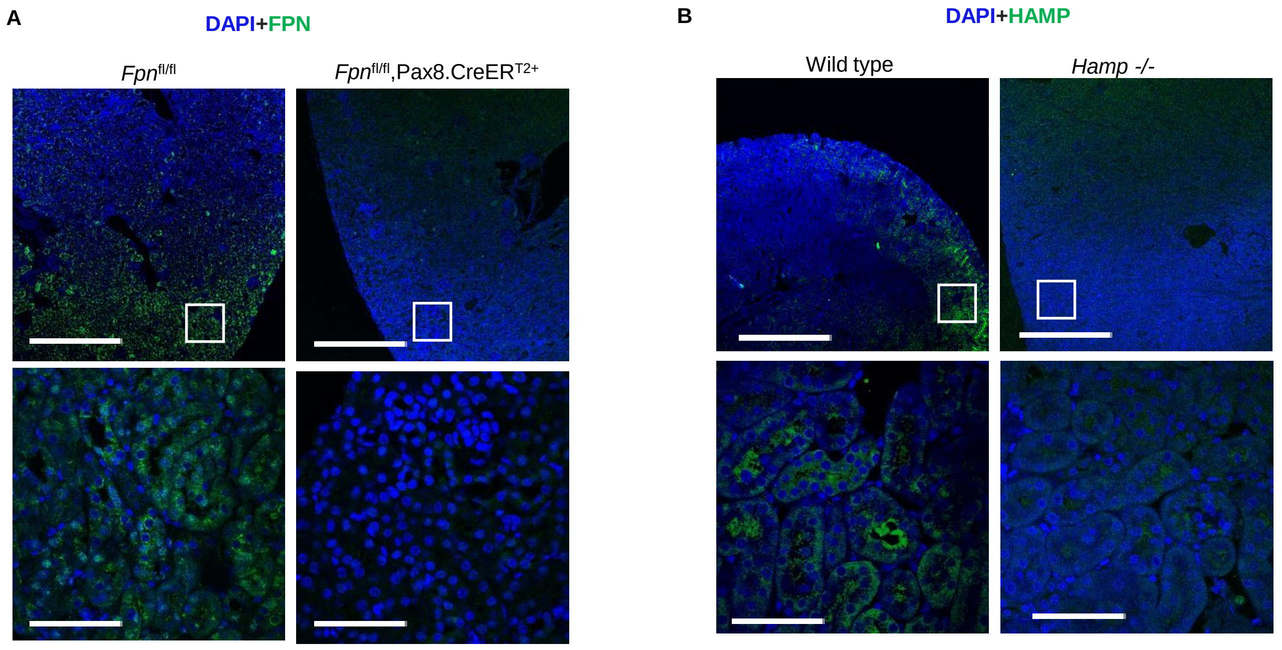

Supplemental figure 1-Confirmation of FPN and HAMP antibody specificity

A. Representative images of FPN immuno-fluorescent staining in the renal cortex of *Fpn*$^{fl/fl}$,Pax8.CreER^{T2+} animals and *Fpn*$^{fl/fl}$ controls at 3 months of age. Top panels, scale bar=200µm, original magnification x10. Lower panels, scale bar=25µm, original magnification x60.

B. Representative images of HAMP immuno-fluorescent staining in the renal cortex of *Hamp -/-* animals and wild type controls at 3 months of age. Top anels, scale bar=200µm, original magnification x10. Lower panels, scale bar=25µm, original magnification x60

Supplemental Figure 2

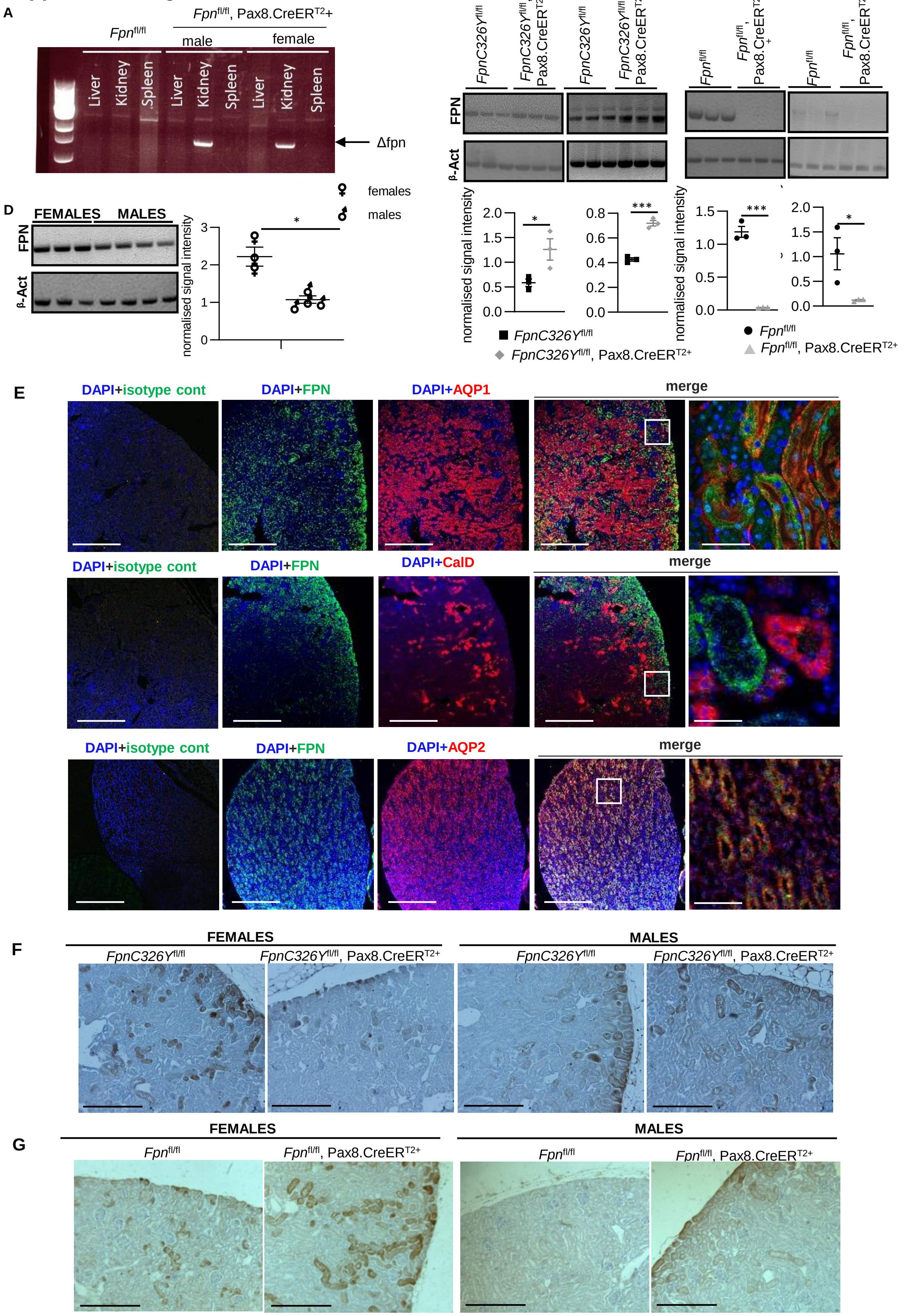

Supplemental Figure 2- Confirmation of Pax8.CreER^{T2+} activity and specificity

A. Genotyping of *Fpn*$^{fl/fl}$ and *Fpn*$^{fl/fl}$,Pax8.CreER^{T2+} mice using genomic DNA extracted from the liver, spleen and kidney at 1 week post tamoxifen induction.

B. Western Blot for FPN in kidneys of female and male *FpnC326Y*$^{fl/fl}$,Pax8.CreER^{T2+} mice and *FpnC326Y*$^{fl/fl}$ controls at 3 months post-tamoxifen treatment. Quantitation of signal intensity shown in bottom panel.

C. Western Blot for FPN in kidneys of female and male *Fpn*$^{fl/fl}$,Pax8.CreER^{T2+} mice and *Fpn*$^{fl/fl}$ controls at 3 months post-tamoxifen treatment. Quantitation of signal intensity shown in bottom panel.

D. Western Blot for FPN in kidneys of wild type females and male littermates at 3 months of age. Quantitation of signal intensity shown in bottom panel.

E-G. Larger versions of panels Fig1A, 1G and 1E respectively.

Values are shown as mean± standard error of the mean. *p<0.05, ***p<0.001.

Supplemental Figure 3

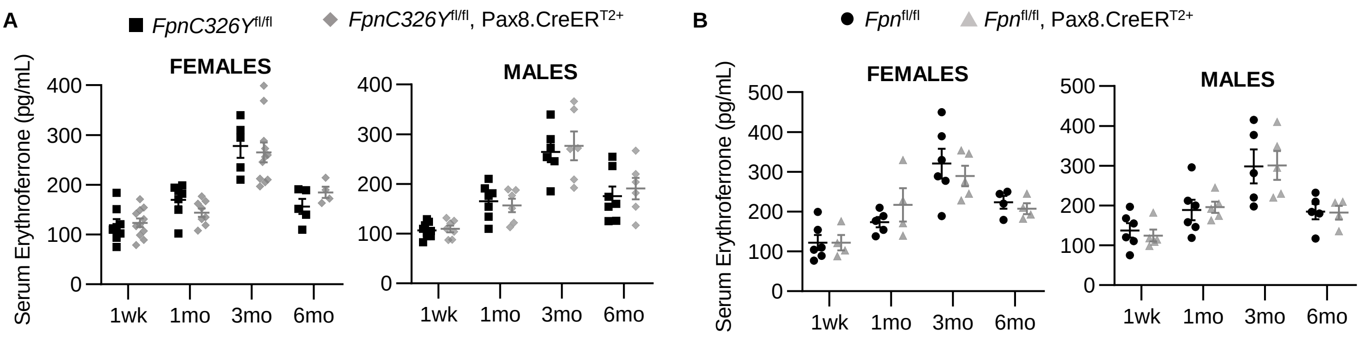

Supplemental figure 3- Serum erythroferrone levels are not altered in *FpnC326Y*$^{fl/fl}$,Pax8.CreER^{T2+} and *Fpn*$^{fl/fl}$,Pax8.CreER^{T2} mice.

A. Serum erythroferrone levels in female and male *FpnC326Y*$^{fl/fl}$,Pax8.CreER^{T2+} mice and *FpnC326Y*$^{fl/fl}$ controls at 1 week, 1 month, 3 months and 6 months post-tamoxifen treatment.

B. Serum erythroferrone levels in female and male *Fpn*$^{fl/fl}$,Pax8.CreER^{T2+} mice and *Fpn*$^{fl/fl}$ controls at 1 week, 1 month, 3 months and 6 months post-tamoxifen induction.

Values are shown as mean± standard error of the mean.

Supplemental Figure 4

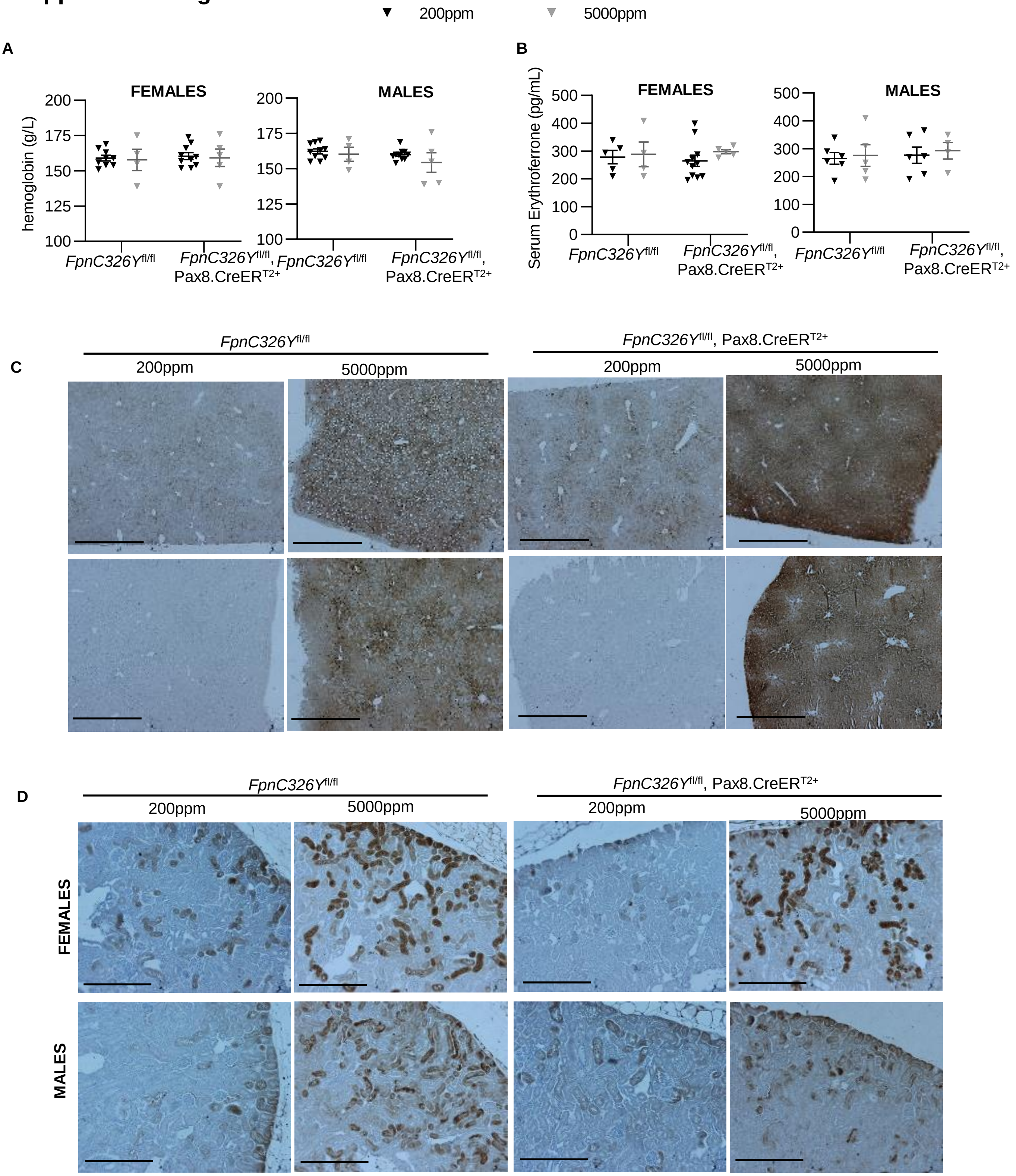

Supplemental figure 4- Haemoglobin and serum erythroferrone levels are not altered in *FpnC326Y*$^{fl/fl}$,Pax8.CreER^{T2+} fed an iron-loaded diet.

A. Haemoglobin levels in female and male *FpnC326Y*$^{fl/fl}$,Pax8.CreER^{T2+} mice and *FpnC326Y*$^{fl/fl}$ controls fed either a control diet (containing 200ppm iron) or an iron-loaded diet (containing 5000ppm iron) from weaning for 3 months.

B. Serum erythroferrone levels in female and male *FpnC326Y*$^{fl/fl}$,Pax8.CreER^{T2+} mice and *FpnC326Y*$^{fl/fl}$ controls fed either a control diet (containing 200ppm iron) or an iron-loaded diet (containing 5000ppm iron) from weaning for 3 months.

Values are shown as mean± standard error of the mean.

C-D. Larger versions of panels Fig3H and 3I respectively.

Supplemental Figure 5

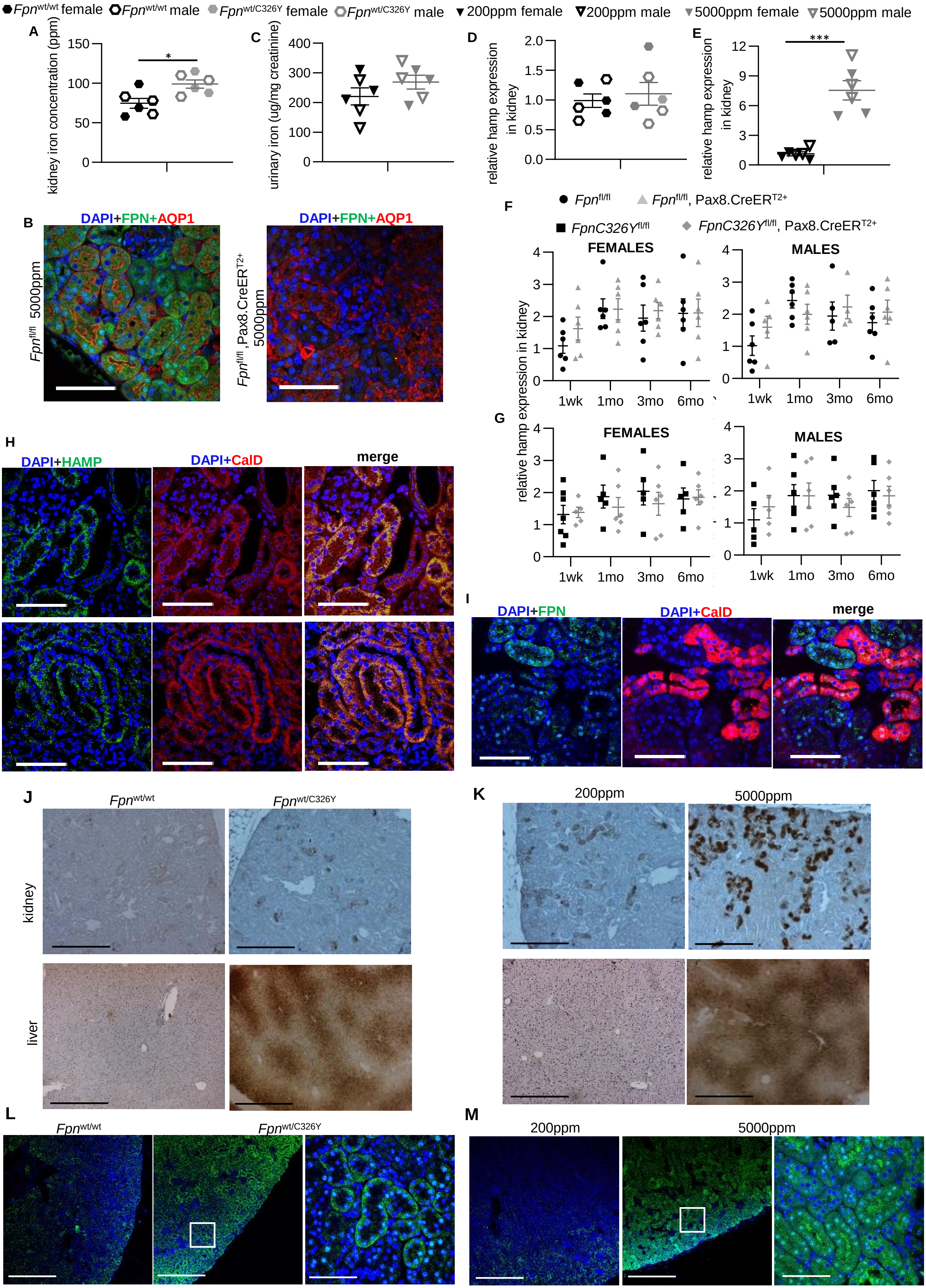

Supplemental figure 5- Renal iron handling and renal *hamp* expression in the different mouse models

A. Renal iron levels in *Fpn*$^{wt/C326Y}$ animals and *Fpn*$^{wt/wt}$ controls at 6 months of age.
B. Representative images of FPN and AQP1 immuno-fluorescent staining in the renal cortex of *Fpn*$^{fl/fl}$,Pax8.CreER^{T2+} animals and *Fpn*$^{fl/fl}$ controls fed an iron-loaded diet containing 5000ppm from weaning for 3 months. Scale bar=25µm, original magnification x60.
C. Urinary iron levels (normalised to urinary Creatinine) in wild type animals fed an iron-loaded diet containing 5000ppm iron, or a control diet containing 200ppm iron from weaning for 3 months.
D. Renal *hamp* gene expression in *Fpn*$^{wt/C326Y}$ animals and *Fpn*$^{wt/wt}$ controls at 3 months of age.
E. Renal *hamp* gene expression in wild type animals fed an iron-loaded diet containing 5000ppm iron, or a control diet containing 200ppm iron from weaning for 3 months.
F. Renal *hamp* gene expression in female and male *FpnC326Y*$^{fl/fl}$,Pax8.CreER^{T2+} mice and *FpnC326Y*$^{fl/fl}$ controls at 1 week, 1 month, 3 months and 6 months post-tamoxifen treatment.
G. Renal *hamp* gene expression in female and male *Fpn*$^{fl/fl}$,Pax8.CreER^{T2+} mice and *Fpn*$^{fl/fl}$ controls at 1 week, 1 month, 3 months and 6 months post-tamoxifen induction.
H. Representative images of HAMP and CalD immuno-fluorescent staining in the renal cortex of wild type animals at 3 months of age. Scale bar=25µm, original magnification x60.
I. Representative images of FPN and CalD immuno-fluorescent staining in the renal cortex of *FpnC326Y*$^{fl/fl}$,Pax8.CreER^{T2+} animals at 1 months of age. Scale bar=25µm, original magnification x60

J-M. Larger versions of panels Fig4C, 4D, 4F and 4G respectively.

Values are shown as mean± standard error of the mean. *$p<0.05$.

Supplemental Figure 6

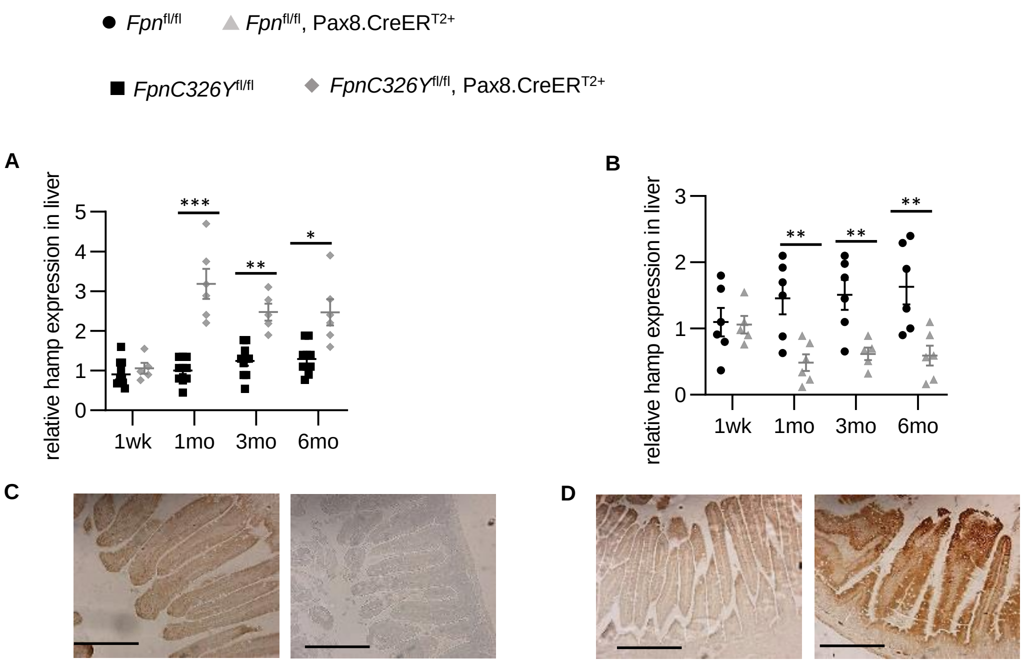

Supplemental figure 6- Liver *hamp* gene expression and gut FPN levels are altered in mice with renal tubule-specific knockout of *fpn* and in mice with renal tubule-specific knock-in of *fpnC326Y.*

A. Relative expression of *hamp* mRNA in livers of female *FpnC326Y*$^{fl/fl,}$ Pax8.CreER^{T2+} mice and *FpnC326Y*$^{fl/fl}$ controls at 1 week, 1 month, 3 months and 6 months post tamoxifen treatment.

B. Relative expression of *hamp* mRNA in livers of female *Fpn*$^{fl/fl,}$ Pax8.CreER^{T2+} mice and *Fpn*$^{fl/fl}$ controls at 1 week, 1 month, 3 months and 6 months post tamoxifen treatment.

C. Representative images of FPN immuno-staining in the gut of female *FpnC326Y*$^{fl/fl,}$ Pax8.CreER^{T2+} mice and *FpnC326Y*$^{fl/fl}$ controls 1 month post tamoxifen treatment. Scale bar=200µm, original magnification x10**.**

D. Representative images of FPN immune-staining in the gut of female *Fpn*$^{fl/fl,}$ Pax8.CreER^{T2+} mice and *Fpn*$^{fl/fl}$ controls 1 month post tamoxifen treatment. Scale bar=200µm, original magnification x10**.**

Values are shown as mean± standard error of the mean. *p<0.05, **p<0.01, ***p<0.001.
